# Dipolar induced spin-lattice relaxation in the myelin sheath: A molecular dynamics study

**DOI:** 10.1038/s41598-019-51003-4

**Published:** 2019-10-15

**Authors:** Felix Schyboll, Uwe Jaekel, Francesco Petruccione, Heiko Neeb

**Affiliations:** 1 University of Applied Sciences Koblenz, RheinAhrCampus Remagen, Remagen, Germany; 2 University of KwaZulu-Natal, Centre for Quantum Technology, Durban, South Africa; 3 Institute for Medical Engineering and Information Processing – MTI Mittelrhein, University of Koblenz, Koblenz, Germany

**Keywords:** Biological physics, Neurology

## Abstract

Interactions between hydrogen protons of water molecules and macromolecules within the myelin sheath surrounding the axons are a major factor influencing the magnetic resonance (MR) contrast in white matter (WM) regions. In past decades, several studies have investigated the underlying effects and reported a wide range of R_1_ rates for the myelin associated compartments at different field strengths. However, it was also shown that the experimental quantification of the compartment-specific R_1_ rates is associated with large uncertainties. The current study therefore investigates the longitudinal relaxation rates within the myelin sheath using a molecular dynamic (MD) simulation. For this purpose, a realistic molecular model of the myelin sheath was employed to determine the dipole-dipole induced R_1_ relaxation rate of the hydrogen protons at clinically relevant field strengths. The results obtained clearly reflect the spatial heterogeneity of R_1_ with a increased relaxivity of myelin water due to a reduced molecular mobility near the membrane surface. Moreover, the calculated R_1_ rates for both myelin water and macromolecules are in excellent agreement with experimental findings from the literature at different field strengths.

## Introduction

Longitudinal relaxation is one of the major contrast mechanisms in the central nervous system and is widely used for the investigation of WM morphology. Even though the underlying effects causing this excellent contrast are a frequently investigated issue in the neuroimaging community, they are still not completely understood. It is well known, for example, that the longitudinal relaxation rate in WM regions depends on local tissue concentration and is strongly correlated with the degree of myelinated axons^1,2^. In these myelin rich areas, approximately 12% of the water molecules occur as thin films with a thickness of 35 nm between the lipid membranes surrounding the axons^3,4^. Even though this so-called myelin water is not visible in all MRI acquisitions, it mixes with the MR-visible free water pool in the intra-/extra-cellular space, thereby affecting the observable R_1_ relaxation rate. Here and in the following, the term “water” refers exclusively to the pool of H_2_O molecules which are bound between the myelin bilayers.

In contrast to the intra-/extra-cellular water, myelin water molecules diffuse predominantly close to the membrane surface and interact with the macromolecules, thereby affecting their R_1_ rate. One important mechanism is the transfer of magnetization between the macromolecular pool and the surrounding water. This magnetization transfer (MT) is mainly governed by two effects, namely the through space dipole-dipole coupling between the hydrogen nuclei and the direct exchange of individual protons or hydroxide groups between water and macromolecules^5–7^. These biophysical and biochemical processes between adjacent compartments are referred to as through space cross-relaxation and chemical exchange, respectively. However, it is controversial and still under discussion to what amount the individual mechanisms contribute to the total magnetization transfer^8^.

Another mechanism that is assumed to play an important role is the reduced diffusivity of water molecules between the lipid membrane due to electrodynamic interactions. The myelin sheath, for example, is composed of various lipid types such as cerebrosides, phospholipids and cholesterol with different tail lengths and head group structures^9,10^. The corresponding spatial variations in length and structure cause a complex and grained membrane surface which constrains the mobility of individual water molecules^11^. In addition, the polar head groups of the phospholipid reduce the diffusivity of water molecules in its immediate neighborhood due to their hydrophilic character. Recent MD simulation studies have shown that this effect is present up to a few nanometers away from the membrane surface and could thus affect the water dynamics over the whole myelin water space^12^. Since the longitudinal relaxation is direct related to the molecular mobility, it is thought that these restrictions strongly affect the R_1_ rate of the myelin water associated molecules^13^.

However, several studies in the past years have shown that an accurate isolation of the MR signal of myelin associated water and a reproducible quantification of its R_1_ rate is quite complicated and associated with uncertainties^13–25^. In addition to the limited accuracy, many of these experiments were performed at different field strengths, which complicates a direct comparison of their findings. On the other hand, in the past decades classical molecular dynamic (MD) simulations have become a reliable and well established tool for modeling the physical properties of molecules and reconstructing experimental data. Moreover, the continuous improvement of molecular force field simulations and the variety of parameterized lipid and protein molecules allows to simulate complex biological structures, i.e. a myelin-alike environment on atomistic scale.

The aim of the current work was therefore the theoretical investigation of the dipole-dipole induced R_1_ relaxation rate in the myelin sheath using an MD simulation. For this purpose, we first carried out an MD simulation of a lipid-water system representing a small fraction of the membrane structure inside the myelin sheath (the lack of protein molecules in this model is discussed in the Limitations section). The hydrogen trajectories obtained from this atomistic simulation were employed in the next step to determine the dipolar induced relaxation rate of the ^1^H protons inside the myelin sheath at field strengths of 1.5 T, 3 T and 7 T. Based on these results, the average R_1_ rates of the lipid membrane and the water compartment were calculated and compared with experimental findings at different field strength. In addition, the cross-relaxation rate between the water and the macromolecules was determined to assess its contribution to the total magnetization transfer.

## Methods

### Dipolar relaxation in a two-compartment system

The formalism for the calculation of nuclear spin relaxation employed in this study is based on the well-established approach introduced by Bloembergen, Purcell and Pound^26^. In this approach the authors derived the relaxation rates in liquid substances for spin-½ particles by treating the motion of the molecules classically whereas the dipolar interaction Hamiltonian was described quantum mechanically. It is assumed that secondary relaxation mechanisms such as *j*–coupling, spin-rotation or chemical shift anisotropy of the molecules are negligible. Under these conditions, the relevant interaction between two equivalent spins *I* and *S* is given by the dipole-dipole Hamiltonian which can be written in terms of second-rank tensors as

1 $${ {\mathcal H} }_{DD}={\sum }_{q=-2}^{2}{\hat{T}}_{2,q}{F}_{q}(r,\,\theta ,\,\phi ),$$

where ${\hat{{\rm{T}}}}_{2,{\rm{q}}}$ are spin operators and *Fq* are second degree spherical harmonics of order *q*. The polar coordinates *r*, *θ* and *φ* describe the position of spin *S* relative to *I*, whereas the main magnetic field, B_0_, defines the $z$-direction of the coordinate system. Spin operators and spherical harmonics satisfy the relationships ${\hat{T}}_{2,-q}={\hat{T}}_{2,q}^{\ast }$ and ${F}_{2,q}={F}_{2,-q}^{\ast }$, respectively, where the asterisk indicates the complex conjugate, and the latter are given by:

2a $${F}_{2,0}=\frac{1-3\,\cos {(\theta )}^{2}}{{r}^{3}},$$

2b $${F}_{2,1}=\frac{\sin (\theta )\cos (\theta ){e}^{-i\phi }}{{r}^{3}},$$

2c $${F}_{2,2}=\frac{\sin {(\theta )}^{2}{e}^{-2i\phi }}{{r}^{3}}.$$

A full definition of the spin operators can be found in^27^. For the case of molecules that are subject to thermal motion, the relative position and thus the Hamiltonian becomes time dependent. Fluctuations with spectral components around the Lamor frequency, ${\omega }_{L}=\gamma {B}_{0}$, serve as energy source for the longitudinal relaxation process. The relevant frequencies for the spin transitions can be obtained from the real part of the corresponding spectral densities which are given by

3 $${J}_{q}(\omega )=\Re ({\int }_{-\infty }^{\infty }{G}_{q}(\tau ){e}^{-i\omega \tau }d\tau )=2{\int }_{0}^{\infty }{\langle {F}_{q}(t){F}_{q}^{\ast }(t+\tau )\rangle }_{t}\,\cos ({\omega }_{}\tau )d\tau ,\,$$

where *G*_*q*_ is the time autocorrelation function of the *q*-*th* spherical harmonic and *τ* is the lag time. The angular brackets on the right-hand side denote the average over time. For isotropic tumbling particles such as water molecules in the liquid phase, the autocorrelation function decays exponentially on timescales of a few picoseconds and the corresponding spectral density can be described by a simple Lorentzian curve. In macromolecules, however, hydrogen nuclei are bonded in a flexible lattice resulting in an overall motion that is governed by the internal vibrations of the nuclei and the global rotation of the whole molecule. Since both processes occur simultaneous and independently on different time scales, the corresponding correlation functions can be factored and written in a normalized form as

4 $$\widehat{{G}_{q}}(\tau )={S}_{\,}^{2}{e}^{-\tau /{\tau }_{M}^{}}+(1-{S}_{\,}^{2}){e}^{-\tau /{\tau }_{i}^{}},$$

where ${\tau }_{M\,}^{\,}\,$is the global correlation time of the macromolecule, ${\tau }_{i\,}^{\,}\,$the internal vibrational correlation time, *S*^2^ the order parameter describing the restriction of the motion of individual protons and can take values from 0 to 1^28^. The respective spectral density can then be written as linear combination as

5 $${J}_{q}(\omega )=2{\mathcal{A}}_{q}\{\frac{{S}^{2}{\tau }_{M}}{1+{\omega }^{2}{\tau }_{M\,}^{\,2}}+\frac{(1-{S}_{\,}^{2}){\tau }_{i}}{1+{\omega }_{\,}^{\,2}{\tau }_{i\,}^{\,2}}\},$$

where ${\mathcal{A}}_{q}$ is the magnitude of the correlation function given by $\langle {F}_{q}(0){F}_{q}^{\ast }{(0+\tau )\rangle }_{\tau }$. Following the semiclassical approach from Bloembergen *et al*., the transition probability per unit time between two two-particle spin states $|{q}_{1}{q}_{2}\rangle$ and $|{q^{\prime} }_{1}{q^{\prime} }_{2}\rangle$, *W*, is given to first order by

6 $${W}_{q}=\frac{1}{t{\hslash }^{2}}{|{\int }_{0}^{t}\langle {q}_{1}{q}_{2}|{{\mathscr{H}}}_{DD}({\rm{t}}{\rm{^{\prime} }})|{q{\rm{^{\prime} }}}_{1}{q{\rm{^{\prime} }}}_{2}\rangle {e}^{-i\omega (q)t{\rm{^{\prime} }}}dt{\rm{^{\prime} }}|}^{2}=\frac{9}{16}{(\frac{{\mu }_{0}}{4\pi }{\gamma }_{I}{\gamma }_{S}\hslash )}^{2}{J}_{q}(\omega ),$$

where $\hslash$ is the reduced Planck constant, *q* = 1,2 is the order of the spherical harmonics and *γ*_*I(S)*_ is the gyromagnetic ratio of spin *I(S)*. Using these definitions and the relationship between the transition probabilities and the longitudinal relaxation rate, which can be derived from the kinetic equations of the state population, the R_1_ rate for two hydrogen nuclei ($I=S=1/2$) can be expressed in terms of the spectral densities as^27^

7 $${R}_{1}=2({W}_{1}+{W}_{2})=\frac{9}{8}{(\frac{{\mu }_{0}}{4\pi }{\gamma }_{H}^{2}\hslash )}^{2}\,[{J}_{1}({\omega }_{L})+{J}_{2}(2{\omega }_{L})].$$

Here, *μ*_0_ is the vacuum permeability and *γ*_*H*_ is the gyromagnetic ratio of the hydrogen nuclei. Eq. (7) holds true for two nuclei in magnetically equivalent environments and can easily be extended for a many spin system. In order to apply Eq. (7) to the myelin sheath, it must be taken into account that the dynamics of hydrogen nuclei within rigid macromolecules is different from those within the mobile water molecules. The motion-restricted nuclei are subject to static magnetic fields from neighbouring spins and ions which make them insensitive to exciting pulses near the Lamor frequency^29,30^. Such an excitation pulses, for example, would depose energy in the water molecules but does not affect the net spin population within the macromolecules. Due to their different absorption properties, it follows that the relaxation processes of hydrogen nuclei assigned to the water-pool (WP) and hydrogen nuclei assigned to the macromolecule-pool (MP) have to be determined separately. For this purpose, Eq. (7) has to be extended to account for the different environments:

8 $${}_{j}{R}_{1,A}=\frac{9}{8}{(\frac{{\mu }_{0}}{4\pi }{\gamma }_{H}^{2}\hslash )}^{2}\,{\sum }_{\begin{array}{c}i=1\\ i\ne j\end{array}}^{\,{N}_{A}}[{J}_{1,ij}({\omega }_{L})+{J}_{2,ij}(2{\omega }_{L})].$$

**Figure 1:**
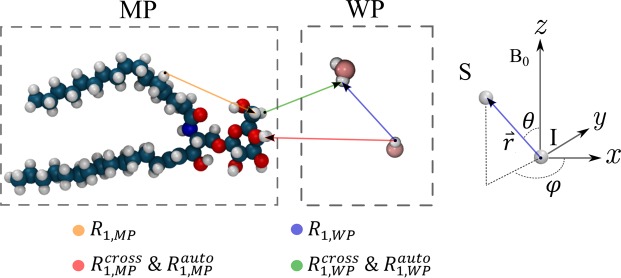
Left: Illustration of the different relaxation mechanism between hydrogen nuclei residing on water and macromolecules; Right: Coordinate system which defines the frame of reference. In this frame, the hydrocarbon tails of the lipids are directed along the x- or y-axis while the main magnetic field points along z.

Here, _*j*_*R*_1,*A*_ is the longitudinal relaxation rate of the *j*-th proton and *N*_*A*_ is the total number of hydrogen protons in pool A (WP or MP), i. e. the hydrogen nuclei are exclusively assigned to either WP or MP. In addition to this compartment-intrinsic induced relaxation, it must be taken into account that the hydrogen nuclei still interact across pools, thereby enhancing their transition probability mutually. Moreover, the unbalanced spin populations due to spectral broadening causes a MT between the two pools. These inter-compartmental interactions, which exclusively involve ^1^H protons from the respective other pool, are referred as auto- and cross-relaxation^31^ and are given by

9 $${}_{j}{R}_{1,A}^{auto}=\frac{1}{16}{(\frac{{\mu }_{0}}{4\pi }{\gamma }_{H}^{2}\hslash )}^{2}{\sum }_{\begin{array}{c}i=1\end{array}}^{\,{N}_{B}}[{J}_{0,ij}(0)+18{J}_{1,ij}({\omega }_{L})+9{J}_{2,ij}(2{\omega }_{L})],$$

10 $${}_{j}{R}_{1,A}^{cross}=\frac{1}{16}{(\frac{{\mu }_{0}}{4\pi }{\gamma }_{H}^{2}\hslash )}^{2}{\sum }_{\begin{array}{c}i=1\end{array}}^{{N}_{B}}[9{J}_{2,ij}(2{\omega }_{L})-{J}_{0,ij}(0)],$$

where *N*_*B*_ is the total number of ^1^H protons assigned to the pool B. Figure 1 shows how the different relaxation processes can be classified in context of the pool affiliation. In order to determine the average auto- and cross-relaxation rates, the Eqs (7–10) have to be averaged over all hydrogen nuclei within the respective pools. For cross-relaxation, this procedure leads to the expression ${R}_{1,A}^{cross}=\frac{1}{{N}_{A}}{\sum }_{j}^{{N}_{A}}{}_{j}{R}_{1,A}^{cross}$, which can be interpreted as the transfer of magnetization per time unit from pool B to A. Since the latter expression corresponds to a double sum over *N*_*A*_ and *N*_*B*_ weighted by the pool size, the average cross-relaxation rates satisfy

11 $${N}_{WP}\,{R}_{1,WP}^{cross}={R}_{1,MP}^{cross}{N}_{MP},$$

which holds also true for the average auto-relaxation rates. To compare the calculated relaxation rates with experimental findings, it has to be considered that the influence of cross-relaxation cannot be distinguished from the influence of chemical exchange in conventional MR experiments. Although both mechanisms gover the transfer of magnetization between different pools, they do not change the sum magnetization of the combined system and can thus be decoupled from the auto- and intrinsic relaxation rates. The effective relaxation rates, ${R}_{1}^{ef}$, for the each of the pool, without the contribution of MT effects, can then be defined as

12a $${R}_{1,WP}^{ef}={R}_{1,WP}+{R}_{1,WP}^{auto},$$

12b $${R}_{1,MP}^{ef}={R}_{1,MP}+{R}_{1,MP}^{auto}$$

It should be mentioned that Eqs (7–10) are expressed in a general form without any assumptions regarding the molecular structure or motion. In the literature it is often assumed that the molecular motion is unrestricted and isotropic. For such a stochastic process, the spherical harmonics yield an exponentially decaying spectral density and the relaxation rates can be expressed in terms of the reorientational correlation time of the water molecules. Since the water molecules are motionally restricted and thus the molecular tumbling is not necessarily isotropic, the general form was used here.

### System preparation and molecular dynamics simulation

To simulate the dynamics of hydrogen atoms inside the myelin sheath, a MD simulation was performed using the GROMACS package 5.1.2 and the CHARMM36 all-atom force field^32,33^. The membrane structure of the myelin sheath was represented by two lipid-leaflets and was modeled according to a realistic myelin alike molecular composition using the CHARMM-GUI web interface^34^. Each leaflet was composed of 27 cholesterol (CHL: C_27_H_46_O), 23 phosphatidylcholines (DPPC: C_40_H_80_NO_8_P), 23 phosphatidylethanolamines (POPE: C_39_H_76_NO_8_P), 23 galactocerebrosides (GalC: C_40_H_77_NO_7_) and 4 sulfatides (GalS: C_40_H_76_NO_11_S). This composition corresponds to a phosphor- and galactolipid content of 46% and 27%, respectively, in accordance with histological observations^10^. The space between the leaflets was solvated with 4699 water molecules, which corresponds to a water mass-ratio of 40%. In addition, 8 sodium molecules were added to the water compartment to neutralize the net charge of the sulfatides. The water molecules between the leaflets were represented by the TIP4p-FB model^35^. This water model yields significantly better results for water specific characteristics compared to the standard mTIP3p model and is, as far as we can judge, compatible with the CHARMM36 force field (see supporting information).

Before the production runs, during which the trajectories were saved for the further analysis, the system was thermodynamically equilibrated for a MD simulation time of 50 ns to body temperature (310.15 K) and normal pressure according the following settings: The equations of motion were solved using the leap-frog algorithm with a simulation time step of 2 fs. The cut-off lengths of the Lennard-Jones potential and short-range Coulomb interaction were set to 12 nm using the Verlet-scheme. Long-range electrostatic interactions were calculated using the particle-mesh Ewald summation with a grid spacing of 0.5 Å. During all simulation steps, the temperature and pressure were kept constant and controlled by the Nosé-Hoover and Parrinello-Rahmen coupling with a time constant of ${\tau }_{T}=1$ ps and ${\tau }_{p}=2\,\,$ps, respectively. The length (but not the angles) of the hydrogen bonds in the membranes was constrained using the LINCS algorithm.

**Figure 2:**
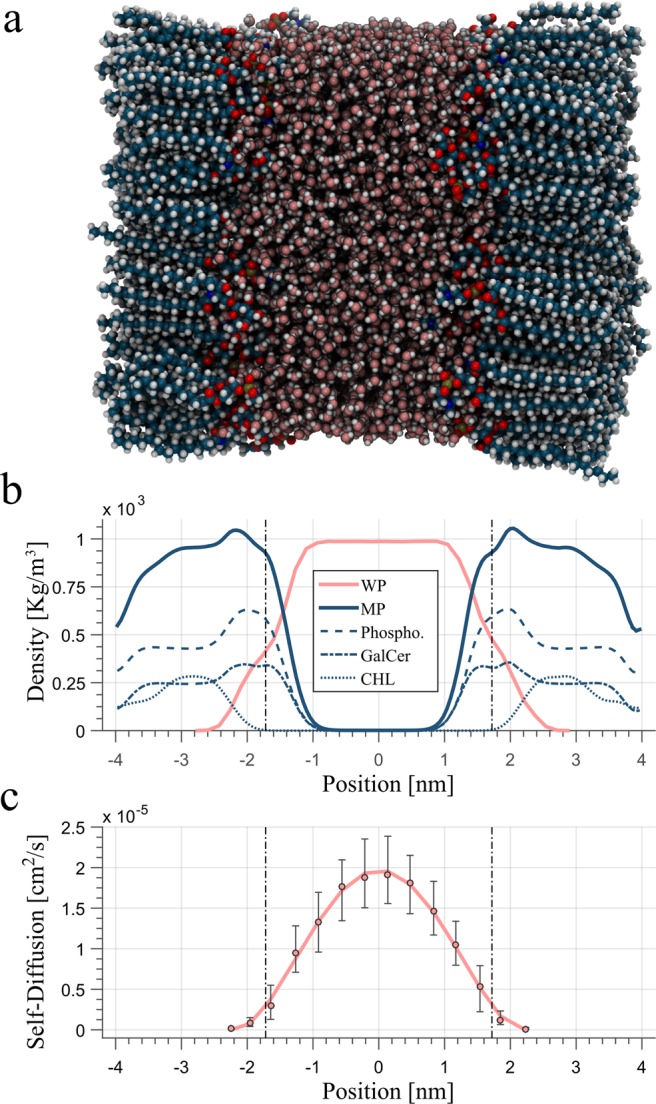
Snapshot of the lipid-water system after the production run and the trajectory conversation (**a**). Density profile of different lipid species inside the membrane as well as water molecules (**b**). Self-Diffusion profile of water molecules across the water pool (**c**). The dotted lines indicate the Gibbs-dividing surface and the error bars the 25^th^ and 75^th^ percentile.

Based on the equilibrated system, the production runs were conducted using the same settings as described above. The run was performed for *T*_*MD*_ = 20 ns and a saving frequency of Δ*T* = 0.5 ps. During the production run, the lipid layers were in the *L*_*β*_ – phase (gel phase) and the tail distance fluctuates around 8.2 nm, which is in accordance with the typical periodicity of the myelin sheath^36,37^. After the run, the trajectories were recomposed again to avoid broken molecule structures due to the periodic boundary condition using the subroutines provided by the GROMACS package. Finally, the trajectories of the hydrogen nuclei of the WP and MP were separately extracted. The final configuration after the trajectory conversion and the density profiles of the corresponding membrane components are shown in Fig. 2a,b.

It should be mentioned that the production run was also performed as an NVE (constant particle number, volume and energy) instead of an NPT (constant particle number, pressure, and temperature) ensemble in order to avoid additional molecular fluctuations due to the temperature and pressure couplings. However, both ensemble configurations yielded, within the standard error, similar relaxation rates for the water molecules. In addition, it should also be mentioned that employing the original water model (mTIP3p) of the CHARMM36 force field instead of the TIP4p-FB model would lead to quite different relaxation rates for the water pool (see supporting information).

### Computation of the relaxation rates

Based on the extracted trajectory sets, the compartment-intrinsic relaxation rates of the WP protons were calculated as follows: First, a single hydrogen nucleus was randomly selected and the relative positions to the remaining hydrogen nuclei (N_WP_ = 9397) within the WP were computed for each time step and converted into polar coordinates. Then, from the polar coordinates, the spherical harmonics given by Eq. (2b,c) and the corresponding correlation functions *G*_1_(*τ*) and *G*_2_(*τ*) were calculated in the interval τ = 0, …, 0.3*T*_*MD*_ using fast Fourier transformation. The truncation at τ = 0.3*T*_*MD*_ was performed to take into account that the tail of the correlation function is dominated by noise due to the finite trajectory length. Next, the spectral densities ${J}_{1}({\gamma }_{H}{B}_{0})$ and ${J}_{2}(2{\gamma }_{H}{B}_{0})$ were calculated for field strengths of 1.5 T, 3 T and 7 T by explicit integration of Eq. (3). Finally, from the spectral densities, the relaxation rate, ${}_{j}{R}_{1,WP}$, of the selected hydrogen nucleus was determined using Eq. (8).

Since the water molecules diffuse several times across the water compartment within *T*_*MD*_ = 20  ns, the calculation above yields representative results for the average relaxation rate of the WP but is not well suited for studying the specific influence of the reduced molecular mobility near the membrane surface. For this purpose, the procedure describe above was repeated for ${T^{\prime} }_{MD}=1.5$ ns, whereby the starting points for the trajectory analysis were randomly selected from the original data set. The time interval of this short-time analysis was chosen so that the average WP-R_1_ rate is comparable with the results where the whole trajectory set was analysed, but the averaged positions of the individual hydrogen nuclei still reflect the location within the WP. In addition to the R_1_ rates, the diffusion coefficients of the water molecules were calculated by fitting the Stokes-Einstein relation to the mean square displacement of the oxygen trajectories. This short-term analysis was performed for 2000 randomly selected trajectory parts and yields the diffusion profile shown in Fig. 2c.

The determination of the relaxation rates of the MP associated hydrogen nuclei (N_MP_ = 13810) was performed similarly, except for the determination of the spectral densities and the short-time analysis. First, the relative positions between a randomly selected hydrogen nucleus and the remaining nuclei were computed and converted into polar coordinates. To determine the spatial distribution of the R_1_ rates within the lipid membrane, the average positions of the selected hydrogen nuclei were stored. Then, the spherical harmonics and the correlation functions were computed using Eqs (2) and (3). Next and in contrary to the calculation for the for the hydrogen nuclei in the WP, the correlation functions were normalized, $\widehat{{G}_{q}}(\tau )={G}_{q}(\tau )/{G}_{q}(0)$, and fitted to Eq. (4) using a least square algorithm. This additional step is necessary since the correlation function of some hydrogen nuclei in the rigid macromolecules does not fully decay to zero within the simulation time of 50 ns. Based on the fit results, the spectral densities ${J}_{1}({\gamma }_{H}{B}_{0})$ and ${J}_{2}(2{\gamma }_{H}{B}_{0})$ and the relaxation rates ${}_{j}{R}_{1,MP}$ were computed using Eqs (6–8). Due to the long computation time of the fitting procedure, the relaxation rates of the MP-protons were calculated for 1381 random selected hydrogen nuclei, which corresponds to 10% of the total compartment size. These hydrogen nuclei were also used to calculate the auto- and cross-relaxation in the MP. For this purpose, the relative positions between one proton and all hydrogen nuclei from the WP were calculated to determine the correlation functions. Since the correlation function describing the inter-compartmental interactions completely converged within the simulation time, the spectral densities ${J}_{0}(0),\,\,{J}_{1}({\gamma }_{H}{B}_{0})$, and ${J}_{2}(2{\gamma }_{H}{B}_{0})$ could be directly computed using explicit integration. Next, from the spectral densities, the auto- and cross-relaxation rates were calculated according Eqs (9) and (10). Then, the computed rates were averaged in order to determine auto- and cross-relaxation rates of the WP using Eq. (11). Finally, the effective relaxation rates, ${R}_{1,WP/MP}^{ef.}$, were determined according Eq. (12) and, in order to compare the results to experimental findings at different field strength, fitted with a least-square algorithm to the function $\,{R}_{1,WP/MP}^{ef.}({B}_{0})={n}_{0}{{B}_{0}}^{n1}$ with parameters *n*_0_ and *n*_1_^38^.

## Results

Table 1 contains the averaged relaxation rates calculated for WP and MP at 1.5 T, 3 T and 7 T. As is evident from the results, all rates increase with decreasing field strength. For ${R}_{1,WP}^{\,}$, the average relaxation rates decrease from 1.175 Hz at 1.5 T by a factor of 1.6 to 0.712 Hz at 7 T. A similar decrease by a factor of 2.0 and 1.7 is also observed for ${R}_{1,MP}$ and ${R}_{1,MP}^{auto}$, respectively. In contrast, for the cross-relaxation rates, a decrease from 0.061 Hz and 0.042 Hz by factor of 3.8 to 0.016 and 0.011 Hz is observed for the WP and MP, respectively. The self-diffusion coefficient of water obtained from the short-term analysis is shown in Fig. 2c. As can be seen in this Figure, diffusion of the water molecules near the membrane surface is significant reduced and reaches a maximum of approximately $2\,\cdot {10}^{-5}$ cm^2^/s in the centre of the WP.

**Table 1:** Averaged relaxation rates for the water and macromolecular pool at different field strengths.

	${{\boldsymbol{R}}}_{{\bf{1}},{\boldsymbol{WP}}}$	${{\boldsymbol{R}}}_{{\bf{1}},{\boldsymbol{WP}}}^{{\boldsymbol{cross}}}$	${{\boldsymbol{R}}}_{{\bf{1}},{\boldsymbol{WP}}}^{{\boldsymbol{auto}}}$	${{\boldsymbol{R}}}_{{\bf{1}},{\boldsymbol{MP}}}$	${{\boldsymbol{R}}}_{{\bf{1}},{\boldsymbol{MP}}}^{{\boldsymbol{cross}}}$	${{\boldsymbol{R}}}_{{\bf{1}},{\boldsymbol{MP}}}^{{\boldsymbol{auto}}}$
*B*_0_ = 1.5 T	1.175	0.061	0.165	4.95	0.042	0.112
*B*_0_ = 3.0 T	0.978	0.039	0.132	3.72	0.027	0.090
*B*_0_ = 7.0 T	0.718	0.016	0.095	2.43	0.011	0.065

**Figure 3:**
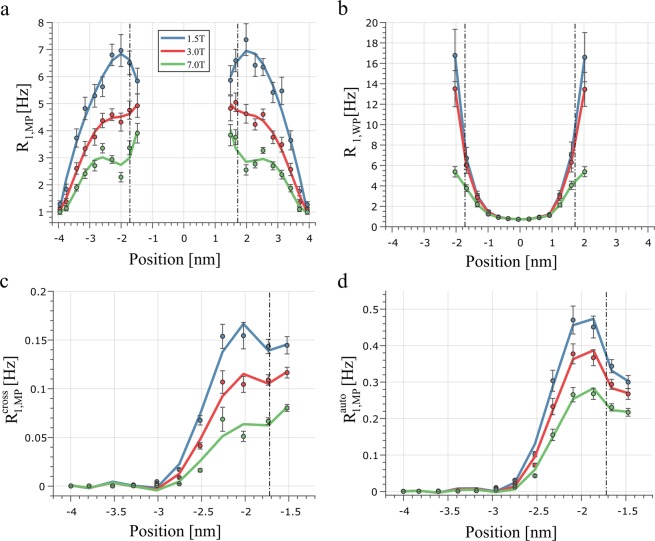
R_1_ relaxation rates in MP (**a**) and WP (**b**) as a function of the proton positions. The macromolecular cross- and auto-relaxation rates are shown in (**c**,**d**), respectively. The error bars indicate the standard error of mean.

Figure 3 shows the different relaxation rates of the WP and MP as a function of the hydrogen positions and the different field strengths. As it is evident from this Figure, the relaxation rates of the macromolecules increases from *R*_1, *MP*_ ≈ 1Hz at the end of the hydrocarbon tails to approximate 5 Hz for 1.5 T, and 4 Hz for 7 T, in the head groups. For the relaxation rates in the WP shown in Fig. 3b, a parabolic relaxation profile is observed with a minimum of *R*_1, *MP*_ ≈ 0.5 Hz at the middle of the WP and a signification increase of ${R}_{1,MP}$ near the macromolecules up to 16 Hz for 1.5 T and 5 Hz for 7 T. Figure 3c,d shows the relaxation profiles of ${R}_{1,MP}^{cross}$ and ${R}_{1,WP}^{auto}$, respectively. Here, the auto- and cross-relaxation rates increase sharply from a few mHz at the tail positions by a factor $\approx 100$ at the head groups, which can be directly attributed to the ${r}_{\,}^{-6}$ dependency of the dipolar interaction between the WP and MP. The fitting procedure of the effective relaxation rates according Eq. (12) yielded the equations ${}_{\,}{}^{\,}R_{1,WP}^{ef}({B}_{0}^{\,})=1.53{B}_{0}^{-0.31}$ for WP and ${}_{\,}{}^{\,}R_{1,MP}^{ef}({B}_{0}^{\,})=5.98{B}_{0}^{-0.45}$ for MP.

## Discussion

The aim of this work was to determine the relaxation rates inside the myelin sheath and its surrounding water compartment using an MD simulation. As is evident from Table 1 and Fig. 3, the calculated relaxation rates clearly reflect the expected R_1_ dispersion in WM structures^38,39^. Moreover, the results obtained demonstrate that the relaxation rate of the myelin water is strongly affected by motional restrictions near the membrane surface. As can be derived from the Stokes-Einstein relation and the relaxation theory, the diffusivity of spherical particles such as water molecules is inverse proportional to the rotational correlation time and thus to the R_1_ rate. This inverse relationship becomes also evident when one compares the spatial distribution of R_1_ rates in water (Fig. 3a) and the spatial profile of the self-diffusion coefficient (Fig. 2c). The influence of the reduced molecular mobility on the R_1_ rate of water is evident over the entire WP and increases the average R_1_ rate in the WP significantly in comparison to water in the liquid phase (1.1 Hz vs 0.3 Hz). In the molecular pool a distinct increase of the relaxation rates along the tail structure was observed at all three field strengths. This behaviour can be attributed to the decreased nuclei mobility in the hydrocarbon chains for lipids in the ${L}_{\beta }$-phase^40^ and illustrates a spatial inhomogeneity of R_1_ within the myelin sheath.

### Relaxation rate

**Figure 4:**
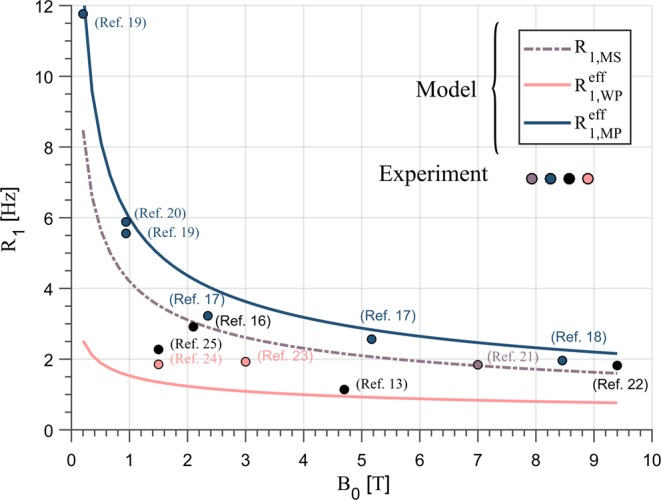
Longitudinal relaxation rates of the WP, MP and the weighted average of the myelin sheath calculated in this study (lines) and the experimental findings (circles) at different field strength. The details of the colorization are described in the main text. The experiments of ref.^16,22,24^ were performed at room temperature between 20° C to 24° C. The remaining data were either *in vivo* experiments or were performed at 39° C (ref.^19^) and 45° C (ref.^18^).

The results of the current work fit well with experimental findings at different field strength^13,16–25^. In^17–20^, phospholipid membranes were extracted from lecithin probes and their R_1_ rates were examined at different temperatures and field strengths between 0.2 T and 8.45 T. For body temperature, the authors reported R_1_ rates between 12 Hz at 0.2 T and 2 Hz at 8.45 T. The function fitted to the effective relaxation rate of the MP as determined theoretically in the current study yields R_1_ rates of 12.4 Hz and 2.3 Hz, respectively, in agreement with experimental findings. In contrast to the measurement on membrane model systems, an experimental quantification of R_1_ within the myelin WP and thus a comparison with the findings of the current study is more complicated. For field strength of 1.5 T, for example, the reported R_1_ rates range between 1.8 HZ and 2.7 HZ (see Table 1 in ref.^41^). These findings are slightly larger than the calculated R_1_ rates of 1.34 Hz at the same field strength in this study. However, it has to be considered that some of these studies employed a two-component (free and bound water) analysis and reported the relaxation rate of the fast decaying component whose relative contribution is between 8–20% as the ${R}_{1}$ of myelin water. This method, however, is not sensitive to the MR invisible macromolecules and as such provides a ${R}_{1}$ rate which contains a signal contribution from the MP due to magnetization transfer. An accurate quantification of R_1_, therefore, includes a proper distinction of the respective molecular compartments_,_ which requires a complex multi-component analysis of the tissue samples. Even if this technique is well understood, the underlying model has several degrees of freedoms or covarying parameters and is therefore associated with systematic uncertainties even in sophisticated experiments. For example, in ref.^21^ the authors combined MT experiments with a multi-echo gradient echo measurement to determine the residence time of water molecules in the myelin sheath at 7 T. Even though this study provided important insights in the WM microstructure, it fails to separate the relaxation rates of two pools and reported an intermediated R_1_ of 1.84 Hz for the myelin sheath. Given the consistent results of the calculated and experimental R_1_ rates for the MP, the reported R_1_ rate can be compared with the average relaxation rate obtained in this work. The latter is given by

13 $$\,{R}_{1,MS}({B}_{0})=\frac{1}{{N}_{MP}+{N}_{WP}}[{N}_{MP}\,{R}_{1,MP}^{ef.}({B}_{0})+{N}_{WP}\,{R}_{1,WP}^{ef.}({B}_{0})]\,$$

where ${R}_{1,MS}({B}_{0})$ denotes the average relaxation rate of the myelin sheath, ${N}_{MP(WP)}$ the hydrogen fractions and ${R}_{1,WP(MP)}^{ef.}({B}_{0})$ the function fitted to the effective relaxation rates. For a field strength of 7 T, Eq. (13) yields an average relaxation rate for the myelin sheath of 1.8 Hz which is very close to the experimental finding. To provide a comprehensive and more illustrative comparison, the reported experimental findings for R1 of the myelin water associated compartment at the different field strength are shown in Fig. 4. The R_1_ rates which could be clearly assigned to the WP, MP or to the weighted average $({R}_{1,MS})$, are illustrated in the corresponding colours. In contrast, the R_1_ rates, which were assigned to the WP but might still contain a signal contribution from the MP were plotted as black circles. As is evident from this Figure, the experimental findings of the MP agree well with the calculated R_1_ rates. In addition, the experimental findings for the myelin water are constrained by the effective relaxation rates of the WP and MP. This means that an appropriate weighting of the respective R_1_ rates as well as the inclusion of magnetization transfer between the pools should allow to reconstruct the experimental data.

### Cross-relaxation and magnetization transfer

Apart from the compartment-specific relaxation rates, the dipolar-induced cross-relaxation rates between WP and MP were calculated. As already mentioned in the introduction, it is still unclear how strong this mechanism contributes to the total magnetization transfer. In saturation-transfer experiments, for example, the magnetization transfer between lecithin/cholesterol and isotopically substituted protons was investigated^42^. The authors concluded that the signal differences between ^3^H protons and ^1^H protons in the surrounding water compartment could be explained by a dominance of magnetically induced exchange over chemical exchange. In contrast, other experiments in tissue and model systems have shown significant pH effects, suggesting chemical exchange^43,44^. If one compares the calculated cross-relaxation rates with results obtained from the measurement of magnetization transfer, this conclusion is also supported by the current study. The total exchange rate, *k*_*WP*_, from the WP to the MP, for example, can be written as

14 $${k}_{WP}={R}_{1,WP}^{cross}+{R}^{ce},$$

where *R*^*ce*^ is the contribution due to chemical exchange. Studies on bovine WM have shown that *k*_*WP*_ is in the order of 12 Hz which is approximately two orders of magnitude higher than the few hundred mHz calculated in this study^16^. This result indicates that the dipole-dipole induced cross-relaxation is negligible compared to chemical exchange and thus plays a minor role for the total magnetization transfer. Unfortunately, and in contrast to the physically well defined cross-relaxation, chemical exchange is characterised by complex chemical (and kinetic) processes on nanosecond-millisecond time scales and requires a comprehensive analysis of the bound enthalpy and the electron configurations of the reactants. Since these parameters are not extractable from a classical MD simulation, a more detailed investigation of the chemical exchange contribution is outside the scope of the current work.

Relating the calculated R_1_ rates within the myelin sheath to the typical exchange rate of approx. 12 Hz as determined in MT experiments reveals that it might be difficult to extract individual compartment-intrinsic R_1_ rates. The magnetisation transfer is fast compared to the relaxation rates in SM and MW. Therefore, the two pools are in rapid exchange so that hydrogen nuclei within the myelin appear as a single magnetisation pool on the longitudinal relaxation time scale.

### Limitations

The current study is subject to some limitations that may have an impact on the interpretation of the reported findings. First, it should be noted that the R_1_ rates were determined for a perpendicular orientation of the membrane normal relative to the main magnetic field (see Fig. 1). As NMR studies have shown, however, the relaxation rate of macromolecules is affected by the membrane orientation relative to B_0_^45,46^. This orientation dependency should also hold true for the R_1_ relaxation rates of the MP as reported in Table 1. Second, it should be mentioned that the membrane model employed here did not consider the protein content inside the myelin sheath. Previous studies have shown that the protein fraction makes up between 19% to 27% of the dry mass^47,48^ of the myelin sheath and consists mainly of myelin basic (∼30%) and proteolipid proteins (∼50%)^49^. Several studies have shown that some of these proteins are located only in the cytoplasmic space or are surrounded by a unique lipid composition^50,51^. The inclusion of proteins employing a realistic localization within the cellular environment and molecular distribution would therefore significantly increase the simulation run-time. However, the absence of proteins might not signifcantly alter the calculated R_1_ rates as can be roughly estimated from the results presented in^18^. Here, the authors investigated the effects of proteins on the R_1_ rate of choline lipids. It was demonstrated that the relaxation rate increases by about 38% for a lipid to protein ratio of 114, which approximately corresponds to the ratio within the myelin sheath. It was suggested that the higher R_1_ rate can be explained by the restriction of the lipid motion due to the heavier protein molecules. However, in the current study calcium pump proteins (∼110 kD) were added to the choline layers, which are approx. five times heavier than the myelin basic or proteolipid proteins (∼18 kD to 30 kD). It can therefore be assumed that the lighter proteins within the myelin sheath might have less influence on the lipid dynamics and thus on the R_1_ of the membrane sheath. This assumption is also supported by a recently published *ex vivo* study, which concluded that the R_1_ relaxation in WM regions is mainly governed by the lipid and not the protein content^52^. Therefore, it can be presumed that a more realistic molecular system including proteins would yield a slightly higher R_1_ rate for the solid myelin but should not significantly affect the final interpretations of this work. Finally, the current study only investigated the effect of the ^1^H induced dipole-dipole relaxation. Although this should be the most effective relaxation mechanism in biological tissue, it can be assumed that other spin = 1/2 particles such as the phosphorus nuclei and secondary effects such as J-coupling, susceptibility anisotropy or the iron content additionally affect the R_1_ rate.

## Conclusion

In this work we investigated the dipolar-induced R_1_ relaxation inside the myelin sheath at several clinical relevant field strengths of 1.5 T, 3 T and 7 T. For this purpose, we employed an atomistic model of the myelin sheath using a realistic lipid composition and a suitable water model. The calculated results are in excellent agreement with experimental findings over a wide range of clinically relevant field strengths. Given the consistent results, the presented model might serve as a basis to investigate further MR properties of the myelin sheath on a microscopic scale. Furthermore, the results obtained suggest that the dipolar induced cross-relaxation plays a minor role for MT and illustrates the increased R_1_ rate of water molecules due to the reduced molecular mobility. Especially the accurate determination of the compartment specific relaxation rates shown in Table 1 might be helpful in future studies to assess experimental findings or to reconstruct the MR signal evolution in myelin rich structures.

## Supplementary information

Supplementary Information for: Dipolar induced spin-lattice relaxation in the myelin sheath: A molecular dynamics study

## Data Availability

The simulation files to generate the MD data are available from the corresponding author on request.
