# Supplementary material for: Dipolar induced spin-lattice relaxation in the myelin sheath: A molecular dynamics study
Source: Sci Rep. 2019 Oct 15;9:14813. doi: 10.1038/s41598-019-51003-4 (PMC6794311; doi:10.1038/s41598-019-51003-4)
Supplement: Supplementary file 1 — Supplementary Information for: Dipolar induced spin-lattice relaxation in the myelin sheath: A molecular dynamics study [file 41598_2019_51003_MOESM1_ESM.docx]

**Supplementary Information for:**

**Dipolar induced spin-lattice relaxation in the myelin sheath: A molecular dynamics study**

Felix Schyboll^1^, Uwe Jaekel^1^, Francesco Petruccione^2^, Heiko Neeb^1 ,3,*^

^1^University of Applied Sciences Koblenz, RheinAhrCampus Remagen, Germany
^2^ University of KwaZulu-Natal, Centre for Quantum Technology, Durban, South Africa

^3^ Institute for Medical Engineering and Information Processing – MTI Mittelrhein, University of Koblenz, Germany

*[neeb@hs-koblenz.de](mailto:neeb@hs-koblenz.de)

**Validation of bulk water and DPPC/POPE parameters simulated with the mTIP3p and TIP4p-FB water model using the CHARMM36 force field.**

The CHARMM36 (C36) force field (ff) was developed for simulating complex molecular structures such as lipids, proteins or amino acids on an atomistic scale and predicts many molecular specific parameters with a remarkable precision. Furthermore, the C36 ff provides a wide range of predefined biomolecules which can easily be combined to realistic biological systems. However, the C36 ff in its standard configuration fails to describe some important water specific characteristics such as viscosity or NMR relaxation rates. This is because C36 ff was originally optimized for the mTIP3p water model which was derived from the traditional three-site TIP3p model. This water models predicts, however, an anomalously high dielectric constant, overestimates the self-diffusion coefficient and fails to describe some other water specific features^1-4^. These discrepancies render the combination of C36 ff and the standard mTIP3p model difficult for studying water-related features such as the relaxation rates in myelin water. Therefore, in order to study R_1_ relaxation within the myelin sheath, the molecular dynamic simulations have to be performed with an alternative water model that describes the hydrodynamical properties more accurately. Such a model was recently developed by systematically adjusting the charge position and the Lennard-Jones potential to a set of experimental quantities^5^. This so called TIP4p-FB model allows to reconstruct a wide range of water specific properties in excellent agreement with experimental findings. Moreover, this model was successfully tested in combination with the C36 ff by solvating DPPC and POPC bilayers in both TIP4p-FB and mTIP3p-simulated water molecules and comparing structural parameters such as the area per lipid, compressibility or the X-ray scattering profile^6^. However, this model is not well established yet and the validation of the some hydrodynamic features are still pending. The aim of this section is therefore to continue the evaluation of the TIP4p-FB water model by extending the list of validated parameters and compare them with corresponding results obtained from the standard water model. For this purpose, MD simulations with both the standard mTip3p and the TIP4p-FB water models were performed to determine the rotational correlation time of the O-H and H-H vector as well as the R1 relaxation rate. Furthermore, to ensure the compatibility of the TIP4p-FB water model with C36 ff, the lateral diffusion coefficient (D*_L_*), the Luzzati-thickness (D_B_) and the hydrocarbon-thickness (D_C_) were calculated for POPE and DPPC lipids.

**Computation of bulk water parameters**

To compute the rotational correlation time and the R1 relaxation rate, MD simulations of 2230 mTIP3p and TIP4p-FB water molecules at 298.15 K were performed using the GROMACS package. For all preparation and simulations steps, the parameters described in the main text were used. The production runs were performed for 50 ns and the trajectories were subsequently recomposed to avoid broken molecules. The relaxation rate of the water molecules was calculated from the hydrogen trajectories for 3 T using Eq. (4) and (7) from the theoretical background section of the main text and following the procedure described in the Method section. To allow for a comparison of the computed R_1_ with previously studies, the relaxation rate was calculated for the inter – and intramolecular dipole-dipole interactions. In addition, to quantify the dynamical properties of the water models, the rotational correlation times, $\tau_{l}^{a}$, of the H-H and O-H vector were computed from the corresponding autocorrelation functions:

$G_{l}^{a}(\tau)=\left\langle P_{l}(\boldsymbol{e}^{a}(t)\cdot\boldsymbol{e}^{a}(t+\tau)) \right\rangle\propto e^{-\frac{| \tau_{l}^{a} |}{\tau}}$,

where $P_{l}$ is a Legendre polynomial of degree $l$, $\boldsymbol{e}^{a}$ is the unit vector along the O-H or the H-H vectors and $\tau$ the lag time. The correlation times were computed for the $l=2$ case by integrating the normalized correlation function , $G_{2}^{a}(\tau)/G_{2}^{a}(0)$, from 0 to 500ps. In addition, the reflecting boundary condition was removed from the trajectory set to determine the predicted self-diffusion coefficient, $D_{H_{2}O}$, for each water model by fitting the Stokes-Einstein relation to the mean square displacement of the oxygen nuclei. The results obtained are summarized in Table S1.

**Computation of DPPC and POPE parameters**

To determine the lipid-related parameters, two bilayers consisting of 64 DPPC and 64 POPE lipids were solvated in 2578 mTIP3p and TIP4p-FB water molecules, respectively. The equilibration steps and the production run were performed at 323.15 K and with the same configuration and interaction parameters described in the main text. Furthermore, the production runs were extended from 50 ns to 100 ns to ensure sufficiently long simulation time for the calculation of the lateral diffusion coefficient and the structural lipid parameters. After the production run, the molecules were recomposed and the periodic boundary condition were removed. Then, the centre of mass motion of each leaflet was removed and the lateral diffusion coefficient of the phosphorous atoms in the lipid headgroups was determined. These steps were performed using the subroutines provided by the GROMACS software package. Next, the Luzzati-thickness, D_B_, was determined from the water distribution by,

$D_{B}=D-\int_{-D/2}^{D/2} \rho_{H_{2}O}(x)dx$,

where$D$ is the repeating system-spacing (see Fig. S1b) and $\rho_{H_{2}O}$ is the normalized volume probability of a water molecule along the bilayer normal^7^. $\rho_{H_{2}O}$ was calculated from the time-averaged histogram of the water molecule locations and subsequently normalized to $\rho_{H_{2}O}(D)=1$. In a similar way, the hydrocarbon-thickness, D_C_, was computed by^8^

$D_{c}=\int_{-D/2}^{D/2} \hat{\rho}_{CH}(x)dx$,

where $\hat{\rho}_{CH}$ is the normalized volume probability of the $CH_{2}$ and $CH_{3}$ groups in the lipid tail and given by $\hat{\rho}_{CH}(x)$=$\rho_{CH_{2}}(x)$+$\rho_{CH_{3}}(x)$. The spatial distribution of POPE lipids and the Luzzati- and hydrocarbon thickness are illustrated in Fig. S1b. Finally, the area per lipid, A_pl_, was determined by dividing the mean area of the bilayer by the number of lipids (n=32) building a leaflet. The results obtained are summarized in Tables S2 and S3.

**Table S1 physical properties of bulk water predicted by the mTIP3p and TIP4p-FB model at 298.15 K.**

| **Parameter** | **mTIP3p** | **TIP4p-FB** | **Experimental** |
| --- | --- | --- | --- |
| $R_{1}^{inter}$ [Hz] | 0.038 | 0.105 | - |
| $R_{1}^{intra}$ [Hz] | 0.054 | 0.189 | - |
| T_1_ =1/( $R_{1}^{inter}$+ $R_{1}^{intra}$) [s] | **10.87** | **3.40** | 3.37-3.57^[9,10]^ |
| $\tau_{1}^{HH}$[ps] | 1.58 | 6.07 | - |
| $\tau_{2}^{HH}$[ps] | 0.71 | 2.62 | 2.0^[11]^ |
| $\tau_{1}^{OH}$[ps] | 1.8 | 6.01 | - |
| $\tau_{2}^{OH}$[ps] | 0.67 | 2.35 | 1.95^[12]^ |
| $D_{H_{2}O}$[10^-5^ cm^-2^ s^-1^] | 6.2 | 2.13 | 2.29^[13]^ |

**Table S2 physical and structural properties of DPPC bilayers simulated with the mTIP3p and TIP4p-FB model at T=323.15 K.**

| **Parameter** | **mTIP3p** | **TIP4p-FB** | **Experimental** |
| --- | --- | --- | --- |
| D*_L_* [10^-8^ cm^-2^ s^-1^] | 8.6 | 4.5 | 8.8-14 ^[14,15]^ |
| A_pl_ [Å^2^] | 60.4 | 60.6 | 62.9-64 ^[7,16]^ |
| D_B_ [Å] | 41.3 | 40.7 | 38.5 ^[7]^ |
| 2D_c_ [Å] | 28.2 | 28.0 | 28.5 ^[7]^ |

**Table S3 physical and structural properties of POPE bilayers simulated with the mTIP3p and TIP4p-FB model at T=323.15 K.**

| **Parameter** | **mTIP3p** | **TIP4p-FB** | **Experimental** |
| --- | --- | --- | --- |
| D*_L_* [10^-8^ cm^-2^ s^-1^] | 5.9 | 4.7 | - |
| A_pl_ [Å^2^] | 58.9 | 58.9 | 56^[17]*^ |
| D_B_ [Å] | 41.4 | 41.9 | 41.3^[17]*^ |
| 2D_c_ [Å] | 30.6 | 30.5 | - |

*Measured at 303K


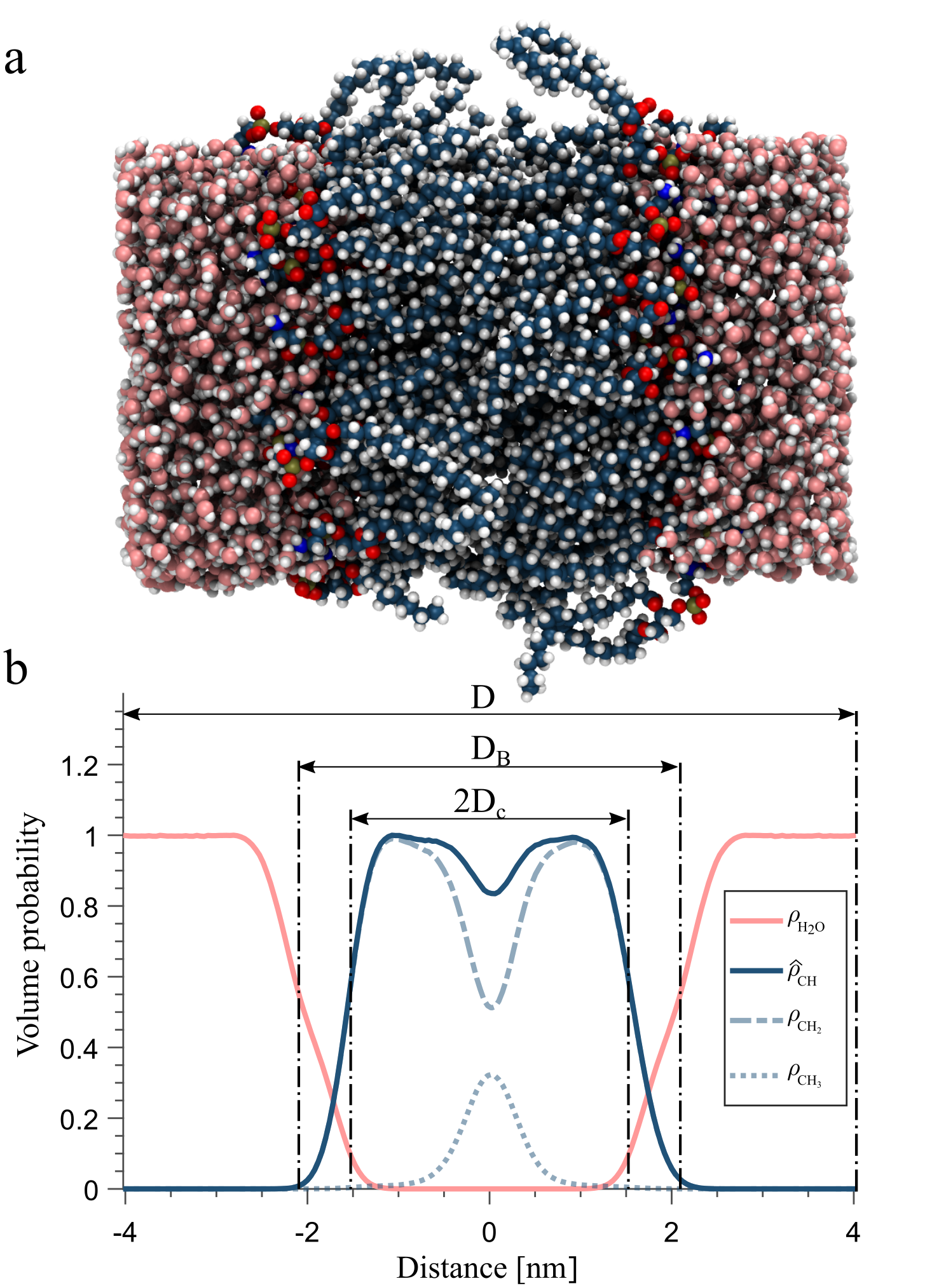


**Fig. S1** DPPC bilayer in the $L_{\alpha}$-phase (fluid-phase) after the production run (a) and the corresponding volume probabilities (b) of the water and the CH-groups of the hydrocarbon tails.

**References:**

1 Braun, D., Boresch, S. & Steinhauser, O. Transport and dielectric properties of water and the influence of coarse-graining: Comparing BMW, SPC/E, and TIP3P models. *The Journal of chemical physics* **140**, 064107 (2014).

2 González, M. A. & Abascal, J. L. The shear viscosity of rigid water models. *The Journal of chemical physics* **132**, 096101 (2010).

3 Calero, C., Martí, J. & Guàrdia, E. 1H nuclear spin relaxation of liquid water from molecular dynamics simulations. *The Journal of Physical Chemistry B* **119**, 1966-1973 (2015).

4 van der Spoel, D., van Maaren, P. J. & Berendsen, H. J. A systematic study of water models for molecular simulation: derivation of water models optimized for use with a reaction field. *The Journal of chemical physics* **108**, 10220-10230 (1998).

5 Wang, L.-P., Martinez, T. J. & Pande, V. S. Building force fields: An automatic, systematic, and reproducible approach. *The journal of physical chemistry letters* **5**, 1885-1891 (2014).

6 Sajadi, F. & Rowley, C. N. Simulations of lipid bilayers using the CHARMM36 force field with the TIP3P-FB and TIP4P-FB water models. *PeerJ* **6**, e5472 (2018).

7 Nagle, J. F. & Tristram-Nagle, S. Structure of lipid bilayers. *Biochimica et Biophysica Acta (BBA)-Reviews on Biomembranes* **1469**, 159-195 (2000).

8 Chiu, S.-W., Pandit, S. A., Scott, H. & Jakobsson, E. An improved united atom force field for simulation of mixed lipid bilayers. *The Journal of Physical Chemistry B* **113**, 2748-2763 (2009).

9 Simpson, J. & Carr, H. Diffusion and nuclear spin relaxation in water. *Physical Review* **111**, 1201 (1958).

10 Krynicki, K. Proton spin-lattice relaxation in pure water between 0 C and 100 C. *Physica* **32**, 167-178 (1966).

11 Halle, B. & Wennerström, H. Interpretation of magnetic resonance data from water nuclei in heterogeneous systems. *The Journal of Chemical Physics* **75**, 1928-1943 (1981).

12 Ludwig, R. NMR relaxation studies in water-alcohol mixtures: the water-rich region. *Chemical physics* **195**, 329-337 (1995).

13 Fennell, C. J. *Development of molecular dynamics techniques for the study of water and biochemical systems*. (University of Notre Dame, 2007).

14 Picard, F., Paquet, M.-J., Dufourc, É. J. & Auger, M. Measurement of the lateral diffusion of dipalmitoylphosphatidylcholine adsorbed on silica beads in the absence and presence of melittin: a 31P two-dimensional exchange solid-state NMR study. *Biophysical journal* **74**, 857-868 (1998).

15 Filippov, A., Munavirov, B., Gröbner, G. & Rudakova, M. Lateral diffusion in equimolar mixtures of natural sphingomyelins with dioleoylphosphatidylcholine. *Magnetic resonance imaging* **30**, 413-421 (2012).

16 Nagle, J. F. *et al.* X-ray structure determination of fully hydrated L alpha phase dipalmitoylphosphatidylcholine bilayers. *Biophysical journal* **70**, 1419-1431 (1996).

17 Rand, R., Fuller, N., Parsegian, V. & Rau, D. Variation in hydration forces between neutral phospholipid bilayers: evidence for hydration attraction. *Biochemistry* **27**, 7711-7722 (1988).
